# The relationship between the COVID-19 anxiety and self-efficacy of patients undergoing hemodialysis: a cross-sectional study

**DOI:** 10.1186/s40359-023-01386-x

**Published:** 2023-10-17

**Authors:** Parvin Mangolian shahrbabaki, Leyla Ahmadi lari, Parniya Abolghaseminejad, Mahlagha Dehghan, Emad Gholamrezaei, Somayeh Zeidabadinejad

**Affiliations:** 1 Nursing Research Center, Department of Critical Care Nursing, Razi Faculty of Nursing and Midwifery, Kerman University of Medical Sciences, Kerman, Iran; 2 MSc in Critical Care Nursing, Department of Nursing, School of Nursing, Larestan University of Medical Sciences, Larestan, Iran; 3 MSc in Health Education & Promotion, Department of Public Health, Sirjan School of Medical Sciences, Sirjan, Iran; 4 Department of Critical Care Nursing, Razi Faculty of Nursing and Midwifery, Kerman University of Medical Sciences, Kerman, Iran; 5 BSc student of Anesthesia, Sirjan School of Medical Sciences, Sirjan, Iran; 6 MSc in Critical Care Nursing, Sirjan School of Medical Sciences, Sirjan, Iran

**Keywords:** Anxiety, Self efficacy, COVID-19, dialysis, Kidney Diseases

## Abstract

**Background:**

The spread of the COVID-19 increased anxiety and stress among patients undergoing hemodialysis. Unpleasant experiences in the lives of these patients reduced their quality of life. Self-efficacy is an effective factor that can improve the quality of life of patients undergoing hemodialysis. Therefore, this study aimed to investigate the relationship between the COVID-19 anxiety and self-efficacy in patients undergoing hemodialysis.

**Methods:**

This cross-sectional, descriptive and analytical study used purposive sampling to select 110 patients undergoing hemodialysis in Sirjan from October to late November 2021. Demographic and clinical information questionnaire, the Corona Disease Anxiety Scale, and the General Self-Efficacy Scale were used to collect data. Data were analyzed using SPSS25. A significance level < 0.05 was considered.

**Results:**

The results showed that the mean score of COVID-19 anxiety was 13.14 ± 7.38, which was lower than the midpoint of the questionnaire. The mean self-efficacy score was 57.72 ± 9.68, with 73.8% (N = 79) of the participants having high self-efficacy and 26.2% (N = 28) having moderate self-efficacy. We found no significant relationship between self-efficacy and the COVID-19 anxiety (P = 0.31), as well as between self-efficacy and the psychological dimension of the corona disease anxiety scale (P = 0.96), but we observed a negative, poor and significant relationship between self-efficacy and its physical dimension (P = 0.048).

**Conclusions:**

Patients undergoing hemodialysis showed low anxiety and high self-efficacy. No significant relationship was found between COVID-19 anxiety and self-efficacy, but a weak correlation was observed with the physical dimension of anxiety. Increasing self-efficacy through patient education and support can improve outcomes and reduce hospitalizations for these patients.

## Introduction

The global health risk presented by the ongoing COVID-19 pandemic is a cause for concern [1]. This pandemic has had a detrimental impact on the physical and mental well-being of individuals, affecting a large number of people worldwide [2]. Additionally, there is uncertainty surrounding when and how this devastating epidemic will conclude, leading to increased anxiety and vulnerability among the population [3]. Reports indicate that individuals with pre-existing conditions like diabetes, kidney failure, and cardiovascular diseases are among the most severely affected and vulnerable groups during this pandemic [4, 5]. The presence of chronic health issues poses significant challenges for healthcare facilities [6, 7].

Patients undergoing hemodialysis typically receive dialysis treatment two or three times per week. However, this treatment regimen exposes them to various situations that further increase their risk of contracting COVID-19 [8]. These situations include close contact with healthcare personnel during dialysis sessions, the use of public transportation or center vehicles, and interactions with other patients and caregivers in waiting areas [9]. As a result, the anxiety levels of these individuals may be heightened during these processes. Studies have shown that the prevalence of anxiety among hemodialysis patients has increased during the pandemic [10–13]. When anxiety is higher than normal, it weakens the body’s immune system, and as a result, the risk of catching the virus increases [14]. Corona anxiety is the anxiety caused by fear of affliction with corona virus [15]. It seems that the reason for corona anxiety is mostly the unknown nature of the virus and cognitive ambiguity, lack of definitive treatment, forced quarantines and economic problems caused by the epidemic [15, 16]. The results of one study has shown that psychological factors such as disease anxiety make a person vulnerable to this virus [17]. For this purpose, it is necessary to extract and examine variables correlated to Corona anxiety so that its negative consequences can be controlled and reduced [18].

Existing research have proved a negative correlation between self-efficacy and negative mental health outcomes resulted from COVID-19 pandemic, such as stress, depression, anxiety and fear [19, 20]. Self-efficacy was defined as the belief in one’s competence and efficiency to successfully tackle tasks by Bandura [21]. Self-efficacy is a fundamental component of the effective self-management of chronic diseases [22]. It is conceptualized as patients’ confidence in their ability to overcome barriers and achieve the desired outcomes [23]. According to self-efficacy theory, if individuals believe that they can achieve a result, they will be a more active participant in the management of their condition, which will facilitate adequate disease control [21]. Self-efficacy plays a vital role in delaying Chronic kidney disease (CKD) progression, and it has been shown that a higher level of self-efficacy in CKD patients is associated with a better quality of life [24] and lower levels of anxiety and depression [25]. Also, it is suggested that higher self-efficacy can prevent poor psychological outcomes during COVID-19 pandemic [26].

According to the above, low self-efficacy usually increases problems and worsens disease-related conditions. Patients with low self-efficacy have a greater chance of developing emotional and social problems, including mental-health conditions such as anxiety and depression [27, 28].

Consequently, it is reasonable to assume that improving and increasing self-efficacy will reduce anxiety under the stress of COVID-19. Notwithstanding, no study to date, to our knowledge, has yielded the relationship between the COVID-19 anxiety and self-efficacy of patients undergoing hemodialysis. So conducting research in this area is essential for improving the overall well-being of hemodialysis patients during the COVID-19 pandemic. By implementing timely therapeutic and care interventions based on the findings, healthcare providers can better support these patients and alleviate their anxiety. Therefore this study was conducted with the aim of the relationship between the COVID-19 anxiety and self-efficacy of patients undergoing hemodialysis.

## Materials and methods

### Study design and setting

This cross-sectional, descriptive correlational study was conducted on the patients undergoing hemodialysis in Imam Reza hospital, Sirjan from October to late November 2021. This hospital is one of the largest medical centers in Kerman province. It is also used as a teaching hospital with 235 active beds.

### Sample size and sampling

Due to the small size of the research population, all patients undergoing hemodialysis in Imam Reza Hospital in Sirjan were examined. 110 patients are undergoing hemodialysis in this hospital. Eligible patients were selected based on the criteria for entering the study and using convenience sampling method. Inclusion criteria included patients aged 18–65 years old, with at least three months of dialysis history [29], who underwent hemodialysis twice a week, were able to speak, read and write Persian, had no obvious cognitive problems, including Alzheimer’s, no stroke and transient ischemic attack, no vision and hearing diseases, no chronic physical disorders such as cardiac, respiratory, hepatic diseases, mental disorders such as severe depression and cognitive disorders, and no history of mental illness or admission to a psychiatric hospital, and exclusion criteria included patients’ experience of a critical incident after the start of the research, admission due to recurrence of the disease, and failure to answer more than a third of the questions. Finally, according to the inclusion criteria, 107 patients were included in the study. And three patients were excluded from the study due to lack of inclusion criteria.

### Measurement

The demographic and background information questionnaire, the Corona Disease Anxiety Scale and the General Self-Efficacy Scale were used in this study.

The demographic and background information questionnaire included information such as age, sex, job, and education.

### Corona disease anxiety scale

The Corona Disease Anxiety Scale (CDAS) was developed and validated by Alipour et al. (2020) in Tehran. The final version of this tool consists of 18 items and 2 components (factors). Items 1–9 measure mental symptoms, while items 10–18 measure physical symptoms. This tool is rated on a 4-point Likert scale (never = 0, sometimes = 1, most of the time = 2, and always = 3). Therefore, the highest and lowest scores in this questionnaire are 0 and 54, with higher scores indicating a higher level of anxiety. The reliability of this tool was obtained for the first factor (α = 0.879), the second factor (α = 0.861) and the whole questionnaire (α = 0.919) using Cronbach’s alpha. Correlation of this tool with the GHQ-28 questionnaire was used to examine the criterion-related validity, and the results showed that the Corona Disease Anxiety Scale with the total score of the GHQ-28 questionnaire and the anxiety component, physical symptoms, impairment in social functioning and depression were 0.483, 0.507, 0.418, 0.333 and 0.269 and all these coefficients were significant at 0.01 level [30].

### General self-efficacy scale

This scale developed by Sherer et al. in 1982 has 17 items rated on a five-point Likert scale (strongly agree, agree, no idea, disagree and strongly disagree). Items 1, 3, 8, 9, 13 and 15 increase from left to right. The scores between 17 and 34 indicate poor self-efficacy, the scores between 34 and 51 indicate moderate self-efficacy, and the scores above 51 indicate very high self-efficacy. The minimum score is 17, while the maximum score is 85, with a higher score reflecting a higher level of self-efficacy. To measure the validity of the instrument, the scores obtained from this scale were correlated with the sizes of several personality traits; the correlation predicted between the General Self-Efficacy Scale and the sizes of personality traits was moderate (0.61) and significant (0.05) [31]. The reliability coefficient of the scale was 0.76 using the Gotman test and 0.79 using Cronbach’s alpha [32]. Barati in Iran checked the validity and reliability of this scale and obtained the correlation of 0.61 from two scales of self-esteem and self-efficacy. The scale reliability was 0.86 using Cronbach’s alpha [33].

### Data collection

In order to collect data, after obtaining the code of ethics and necessary permits from Kerman University of Medical Sciences, as well as after coordinating with the officials of Imam Reza Hospital, the researcher went to the research environment and started sampling. The researcher then explained the objectives of the research to the patients and obtained oral informed consent from eligible patients. The patients were asked to complete demographic information questionnaires, the general self-efficacy scale, and the corona disease anxiety scale accurately. To ensure the comfort of the patients, the questionnaires were provided at least one hour after the start of dialysis, allowing them to answer the questions with full preparation. However, some patients experienced pain and discomfort in their hands due to being connected to the hemodialysis machine, which made it difficult for them to answer the questions. In such cases, the researcher requested the assistance of the patient’s companion or one of the nurses to conduct an interview and fill out the questionnaire on behalf of the patient. To minimize potential biases, the researcher refused to conduct interviews with the patients directly. Instead, they emphasized to the patient or the nurse that the questions should be read in their entirety to the patient, and the patient’s answers should be recorded in the questionnaire. Finally, all the questionnaires were collected and analyzed.

### Data analysis

SPSS-25 was used for data analysis. Descriptive statistics, such as frequency, percentage, mean, and standard deviation, were used to describe the characteristics of the research units. The mean and standard deviation were specifically used to determine the anxiety scores related to COVID-19 and self-efficacy. To investigate the relationship between anxiety related to COVID-19 and self-efficacy among hemodialysis patients, a Pearson correlation test was employed. This test helps determine the strength and direction of the relationship between two continuous variables.

Furthermore, to examine the relationship between anxiety related to COVID-19 and the demographic characteristics of the patients, several tests were used. The Mann-Whitney test, independent t-test, analysis of variance (ANOVA), and Kruskal-Wallis test were utilized. These tests are appropriate for comparing groups or variables with different levels or categories. Similarly, to determine the relationship between self-efficacy and the demographic characteristics of the patients, independent t-tests, Kruskal-Wallis tests, and ANOVA were used. These tests help assess whether there are significant differences in self-efficacy scores across different groups or categories. A significance level < 0.05 was considered.

## Results

**Fig. 1:**
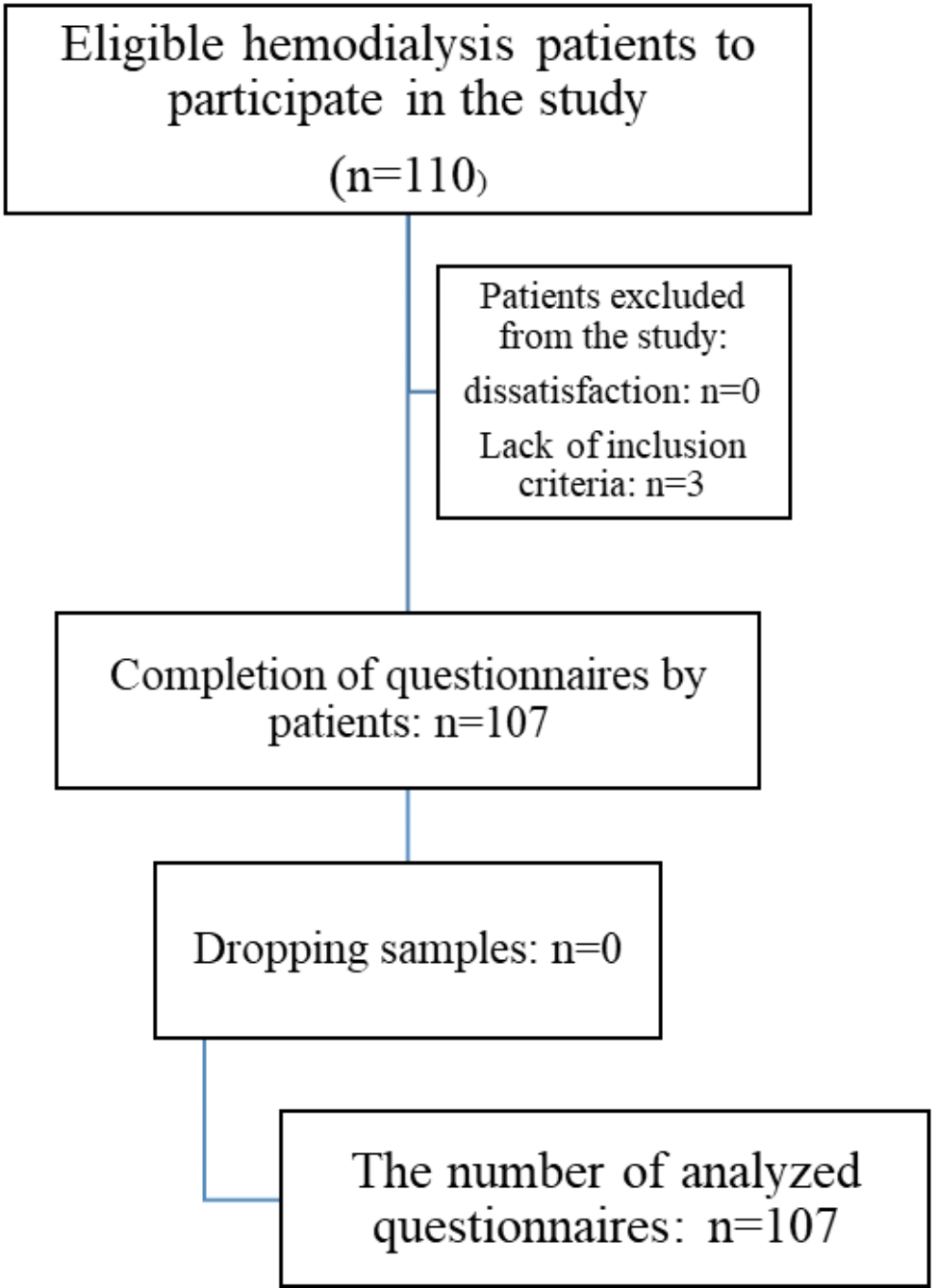
Study flow diagram

In this study, a total of 110 patients initially participated. 3 patients were excluded due to lack of Inclusion criteria. Therefore, the final analysis was conducted on 107 patients (Fig. 1). The study results showed that the mean age of the samples was 52.50 ± 11.08 years. Almost half of the samples were female and had less than a diploma degree; 37.4% of the samples were unemployed/housewives. Most of the samples were married and they had more than 4 children. The majority of the samples had a poor economic status. Hypertension, diabetes or both were the causes of kidney failure in most cases. 72.9% of the samples had no history of kidney transplant. 54.2% of them had a history of heart diseases and 42.1% had a history of diabetes. The majority of patients had no history of addiction, mental disorders and other chronic diseases. 44.9% of patients did not have any physical activity (Table 1).

**Table 1:** Participant’s characteristics and their relation with COVID-19 anxiety and self-efficacy

Variable	N (%)	Anxiety		Statistical test (P value)	Self-efficacy		Statistical test (P value)
		Mean	SD		Mean	SD	
Sex							
Female	52 (48.6)	14.25	8.33	Z = -1.17 (0.24)	56.44	10.28	t = -1.33 (0.18)
Male	55 (51.4)	12.09	6.25		58.93	9.01	
Age (yr.)							
20–40	18 (16.8)	10.78	9.67	F = 1.19 (0.31)	59.93	7.85	H = 4.94 (0.08)
41–60	57 (53.3)	13.84	6.52		58.77	8.50	
61–72	32 (29.9)	13.22	7.34		54.61	11.9	
Education							
< Diploma	52 (48.6)	13.31	6.33	H = 3.93 (0.14)	55.86	10.77	F = 2.06 (0.13)
Diploma	28 (26.2)	10.64	6.55		58.75	8.65	
> Diploma	27 (25.2)	15.41	9.30		60.23	7.90	
Job							
Employed	38 (35.5)	12.45	7.11	F = 0.31 (0.73)	59.97	8.19	F = 3.99 (0.02)
Unemployed	40 (37.4)	13.78	8.47		54.41	9.66	
Retired	29 (27.1)	13.17	6.18		59.35	10.54	
Marital status							
Married	101 (94.4)	13.48	4.84	t = -2.01 (0.047)	57.25	9.58	t = 2.09 (0.04)
Single	6 (5.6)	7.33	4.84		65.62	8.54	
Number of children							
0	13 (12.1)	13.23	9.74	F = 0.34 (0.85)	58.75	9.51	F = 3.04 (0.02)
1	24 (22.4)	11.83	8.73		57.50	8.85	
2	23 (21.5)	13.0	6.43		60.92	8.93	
3	18 (16.8)	13.22	6.10		60.76	6.81	
≥ 4	29 (27.1)	14.24	6.71		53.02	11.11	
Economic status							
Poor	58 (54.2)	12.69	7.48	F = 0.76 (0.47)	56.12	9.94	F = 2.37 (0.10)
Moderate	41 (38.3)	13.20	7.04		60.27	9.45	
Good	8 (7.5)	16.12	8.59		56.25	6.55	
Cause of renal failure							
Hypertension	27 (25.2)	14.85	8.79	F = 0.71 (0.62)	60.0	8.92	F = 2.10 (0.07)
Diabetes	19 (17.8)	14.37	7.50		61.84	10.55	
HTN & Dm	27 (25.2)	11.89	5.90		54.63	8.95	
Kidney stone	11 (10.3)	12.64	5.70		57.95	9.0	
Kidney stone & HTN	9 (8.4)	11.33	6.56		53.06	11.73	
Others	14 (13.1)	12.14	8.69		56.52	8.49	
History of renal transplantation rejection							
No	78 (72.9)	12.47	7.82	t = -1.54 (0.13)	58.52	9.32	t = 1.41 (0.16)
Yes	29 (27.1)	14.93	5.78		55.56	10.48	
History of cardiovascular disease							
No	49 (45.8)	13.24	8.01	t = 0.13 (0.89)	58.49	9.47	t = 0.76 (0.45)
Yes	58 (54.2)	13.05	6.88		57.07	9.90	
History of Diabetes							
No	62 (57.9)	13.55	8.20	Z = -0.19 (0.85)	57.98	9.26	t = 0.33 (0.74)
Yes	45 (42.1)	12.58	6.11		57.36	10.34	
History of addiction							
No	83 (77.6)	13.71	7.91	Z = -1.16 (0.24)	58.49	9.81	t = 1.54 (0.13)
Yes	24 (22.4)	11.17	4.75		55.05	8.93	
History of mental disorders							
No	97 (90.7)	13.08	7.47	t = -0.25 (0.80)	57.40	9.77	t = -1.08 (0.28)
Yes	10 (9.3)	13.70	6.73		60.88	8.58	
History of other chronic diseases							
No	81 (75.7)	13.11	7.58	t = -0.07 (0.94)	58.22	8.97	t = 0.95 (0.34)
Yes	26 (24.3)	13.23	6.85		56.15	11.70	
Physical activity							
No	48 (44.9)	13.52	8.21	F = 0.60 (0.55)	53.62	8.40	F = 10.68 (< 0.001)
Sometimes	39 (36.4)	13.51	7.15		59.65	9.93	
Regularly	20 (18.7)	11.50	5.59		63.81	7.92	

SD: Standard deviation, Z = Mann-Whitney u test, t = independent t test, F = Analysis of variance, H = Kruskal- Wallis H test

Table 2 indicated that the highest mean scores of the anxiety items belonged to “I am worried about the spread of COVID-19 infection to the people around me.” “The COVID-19 anxiety has disrupted my activities.”, “The media’s attention to Corona worries me.”, “I am afraid of catching Corona.” and “My physical activity has decreased due to the fear of Coronavirus.” (Table 2).

**Table 2:** Participants with COVID-19 related anxiety

Item	Mean	SD
Thinking about Coronavirus makes me anxious.	0.87	0.78
I feel stressed when I think about the threat of Corona.	0.85	0.84
I am very worried about the spread of corona disease.	0.97	0.80
I am afraid of catching corona.	1.17	0.88
I think I might catch corona at any moment.	0.67	0.79
With the smallest symptoms, I think I have caught corona virus, so I check myself.	0.62	0.73
I am worried about the spread of corona to people around me.	1.83	0.75
The COVID-19-related anxiety has disrupted my activities.	1.29	0.72
The media’s attention to Corona worries me.	1.18	0.81
Thinking about Corona has disturbed my sleep.	0.41	0.62
Thinking about Corona has made me lose my appetite.	0.40	0.71
I get a headache when I think about corona.	0.35	0.68
I feel like my body is vibrating when I think about corona.	0.25	0.58
I get goose bumps when I think about corona.	0.21	0.51
Corona has become a nightmare for me.	0.39	0.66
My physical activity has decreased due to the fear of corona,	1.12	0.77
It is difficult for me to talk about corona with others.	0.84	0.76
I get heart palpitations when I think about corona.	0.46	0.67

The study results indicated that the mean COVID-19 anxiety was 13.14 ± 7.38, which was lower than the midpoint of the questionnaire [27]. The mean score of the mental dimension was higher than that of the physical dimension. The mean self-efficacy score was 57.72 ± 9.68; 73.8% (N = 79) of the samples had high self-efficacy, while 26.2% (N = 28) had moderate self-efficacy. We found no significant relationship between self-efficacy and the COVID-19 anxiety (P = 0.31), as well as between self-efficacy and mental dimension of the corona disease anxiety (P = 0.96), but we observed a negative, poor and significant relationship between self-efficacy and the physical dimension of the corona disease anxiety (P = 0.048). In other words, the higher the physical anxiety, the lower the self-efficacy and vice versa (Table 3).

**Table 3:** Correlation between COVID-19 anxiety and self-efficacy

Variable	Mean	SD	Self-efficacy	
			Pearson correlation	P value
COVID-19 Anxiety	13.14	7.38	-0.1	0.31
Mental	9.08	4.43	0.005	0.96
Physical	4.06	3.96	-0.19	0.048
Self-efficacy	57.72	9.68		

The study results indicated that married people had a significantly higher level of anxiety than single people. We observed no significant relationship between other background information and the COVID-19 anxiety (Table 1). Married people had a significantly lower level of self-efficacy than single people. Post hoc Bonferroni test showed that unemployed individuals and housewives had lower self-efficacy than employed people (P = 0.03), but self-efficacy was not different between retired, employed, and unemployed individuals. People with four or more children had lower self-efficacy than people with two children (P = 0.03). In addition, people with no physical activity had lower self-efficacy than people who occasionally or regularly did physical activity (P < 0.001).

## Discussion

The present study aimed to investigate the relationship between the COVID-19 anxiety and self-efficacy in patients undergoing hemodialysis. The study results showed that the mean COVID-19 anxiety in patients undergoing hemodialysis was lower than the midpoint and the mean score of the mental dimension was higher than that of the physical dimension. Dehghan et al. (2021) found that 10.4% of patients had severe anxiety, 18.1% had moderate anxiety, and 71.5% had mild anxiety [34]. Their study was consistent with the results of the current study showing the low effect of coronavirus on the anxiety level of patients undergoing hemodialysis in stressful conditions, but Shi et al. (2021) and Hao et al. (2022) disagreed with us and indicated high level of anxiety among patients undergoing hemodialysis during the COVID-19 pandemic [35, 36]. This inconsistency may be because different countries used different isolation measures to reduce the spread of COVID-19. In addition, diverse norms, beliefs, cultural and religious values in countries may affect the anxiety state of patients and different studies used different scales and healthcare systems. Furthermore, at the beginning of the COVID-19 pandemic, patients were more afraid and anxious due to the unknown nature of the disease and its high contagiousness, the lack of awareness and information about how to deal with and treat it. Recently, patients are less anxious due to vaccination and decreased rate of infection and death. Lv et al. (2022) found that high anxiety symptoms in patients undergoing hemodialysis decreased from 40% at the beginning of the COVID-19 outbreak to 28% one year later [37]. However, patients undergoing hemodialysis experience a lot of stress and the COVID-19 may not have a significant effect on increasing their anxiety and stress levels. Depression and anxiety are the most common psychological complications among patients undergoing hemodialysis due to many problems associated with this disease, including loss of freedom, life expectancy, energy, job, income, and sexual ability [34]. Therefore, we require more studies on anxiety of the patients undergoing hemodialysis and various factors affecting it.

The study results showed that 73.8% of the patients had high self-efficacy, while 26.2% had moderate self-efficacy. Oktarina et al. (2019) and Pakaya et al. (2021) supported this result and reported that the majority of patients had high self-efficacy [38, 39], but some studies indicated a moderate level of self-efficacy in patients undergoing hemodialysis [40–43]. Noghani et al. (2020) reported low level of self-efficacy among patients [44], which was contrary to our results because of cultural and geographical differences, sample size, study method and measuring tool.

Self-efficacy plays a central role in motivational processes and acquisitions of the individuals and determines how much effort a person needs for a task. A person with strong self-efficacy will try hard to become successful, while people with poor self-efficacy stop trying after starting any action [45]. A person with high self-efficacy will solve obstacles and problems with higher motivation and will show more efficiency. People with poor self-efficacy believe that things are more difficult than they really are. Self-efficacy beliefs are among the strongest predictors of individuals’ behaviors. Belief in self-efficacy affects many aspects of life such as goal setting, decision making, level of effort, level of continuity, sustainability and struggle with challenging issues, level of motivation, and implementation of goals [46].

Patients should be key members of the care team in the management of chronic diseases. Self-efficacy of patients receiving dialysis is a valuable determinant for effective management, better nursing interventions and outcomes. Evidence suggests that patients receiving dialysis with good self-efficacy reported better outcomes than patients with poor self-efficacy. In addition, self-efficacy mediates the relationship between knowledge and self-care in patients with chronic kidney disease [47].

The results indicated no significant relationship between self-efficacy and the mental dimension of the COVID-19 anxiety but a negative, poor and significant relationship between self-efficacy and the physical dimension of the COVID-19 anxiety, so the higher the physical anxiety, the lower the self-efficacy and vice versa. According to available evidence, no studies specifically investigated the relationship between the COVID-19 anxiety and self-efficacy among patients undergoing hemodialysis and similar studies investigated it at a time other than the COVID-19 outbreak. For example, Lai et al. (2021), Fallah et al. (2019) and Khoshnazar et al. (2014) showed no significant relationship between anxiety and self-efficacy in patients undergoing hemodialysis [48–50], which is almost consistent with the results of the present study. These results contrast with other studies that showed self-efficacy could improve patients’ health because it enabled them to perform their tasks, roles and manage their emotions [51–53]. However, studies with a larger sample size could confirm whether the COVID-19 anxiety is an independent determinant of self-efficacy in patients undergoing hemodialysis.

The study results reported that married people had a significantly higher level of anxiety than single people but found no significant relationship between other background information and the COVID-19 anxiety. Our results were contrary to the results of Al Naamani et al. (2021) and Dehghan et al. (2021), who found that female patients undergoing hemodialysis had the highest level of anxiety [34, 54], which might be due to their concern about infection of their family members during the COVID-19 outbreak. However, Al Naamani indicated no relationship between age, marital status, education level, occupation and anxiety [54]. Several other studies found that monthly income, education, employment status, insurance, duration of dialysis, pandemic publicity, and physical condition were closely related to high anxiety during the COVID-19 outbreak [36, 55]. In addition, previous studies found that lower/upper secondary education was a risk factor for depression in hemodialysis patients [56, 57]. Individuals with higher levels of education can process stressful information more scientifically, participate in organized social activities to protect themselves from psychological stress [57], and seek professional care when facing a psychological condition. Awareness of the seriousness of the epidemic is a protective factor against depression. People who felt the seriousness of COVID-19 were more likely to seek information about it [58].

Married individuals in the present study had a significantly lower level of self-efficacy than single individuals; unemployed individuals and housewives had less self-efficacy than employed people, but self-efficacy was not different between retired, employed, and unemployed individuals. People with four or more children had lower self-efficacy than those with two children. In addition, people with no physical activity had less self-efficacy than those who occasionally or regularly did physical activity. Qalawa et al. (2022) showed a significant relationship between socio-demographic characteristics (gender, age, marital status, occupation and education level) and chronic disease self-efficacy in patients undergoing hemodialysis [59]. Almutary et al. (2021) reported no significant relationship between age, gender and self-efficacy but observed significant relationship between employment status and perceived self-efficacy related to the disease [23]. Some other previous studies also confirmed the relationship between employment status and self-efficacy [60–62], which may be due to the relatively higher socioeconomic status, the availability of more financial resources and a socially stable status of the employed people. Almutary demonstrated a correlation between the type of dialysis and self-efficacy, so patients undergoing peritoneal dialysis had more perceived self-efficacy related to illness than patients undergoing hemodialysis [23]. Their result is consistent with another study in which receiving peritoneal dialysis contributed to greater self-efficacy than receiving a hemodialysis [63]. Patients receiving peritoneal dialysis are thought to be more independent because most of them manage peritoneal dialysis on their own. Another study showed that patients receiving peritoneal dialysis had higher scores for positive attitude, stress reduction and decision-making than patients undergoing hemodialysis [64]. We only studied patients undergoing hemodialysis, so further studies are necessary to confirm the relationship between the type of dialysis and self-efficacy.

The findings of this study have important implications for healthcare professionals working with hemodialysis patients, particularly in relation to reducing COVID-19 anxiety. The results provide valuable insights that can guide the development and implementation of supportive interventions tailored to the specific needs of these patients. By utilizing these findings, healthcare professionals can effectively address and alleviate COVID-19 anxiety in hemodialysis patients, ultimately improving their overall well-being and quality of care. This study also had limitations. One limitation of this study is the small size of the researched population, which calls for caution when generalizing the results to all patients. Additionally, the study has other limitations, such as the reliance on self-reported questionnaires. This introduces the possibility of inaccurate and dishonest responses from patients. Moreover, some patients expressed impatience during hemodialysis and wanted to take the questionnaires with them to complete in the next session. However, to ensure the integrity of the results, the researcher kindly requested that patients complete the questionnaires by the end of their hemodialysis time, despite the potential negative impact on sampling time.

## Conclusion

The study results showed that the patients undergoing hemodialysis had a low level of anxiety and high self-efficacy. We found no significant relationship between the COVID-19 anxiety and self-efficacy but only a weak correlation with the physical dimension of anxiety. Patients with chronic kidney disease are undergoing prolonged hemodialysis treatment, and depression and anxiety are common among these patients, which affect their quality of life, so self-efficacy is effective in controlling and achieving the outcomes expected by the patients. Therefore, increasing self-efficacy of patients can increase their self-care ability. As a result, patient education and support is necessary to increase their self-efficacy, improve outcomes and reduce unnecessary hospitalizations. Therefore, it is suggested that health care workers take effective steps towards the overall well-being of these patients by designing and implementing supportive interventions. It is also suggested that researchers conduct this study on a larger scale in order to achieve more accurate results.

## Data Availability

All data generated or analysed during this study are included in this published article.
